# Non-biological Complex Drugs (NBCDs): Complex Pharmaceuticals in Need of Individual Robust Clinical Assessment Before Any Therapeutic Equivalence Decision

**DOI:** 10.3389/fmed.2020.590527

**Published:** 2020-11-23

**Authors:** Rogério Sá Gaspar, Beatriz Silva-Lima, Fernando Magro, Armando Alcobia, Fernando Leal da Costa, José Feio

**Affiliations:** ^1^Departamento de Sócio Farmácia, Faculdade de Farmácia, Universidade de Lisboa, Lisboa, Portugal; ^2^Institute for Biosciences and Bioengineering (iBB), Instituto Superior Técnico, Universidade de Lisboa, Lisboa, Portugal; ^3^Departamento de Ciências Farmacológicas, Faculdade de Farmácia, Universidade de Lisboa, Lisboa, Portugal; ^4^Research Institute for Medicines (iMed), Faculdade de Farmácia, Universidade de Lisboa, Lisboa, Portugal; ^5^Department of Biomedicine, Unit of Pharmacology and Therapeutics, Faculty of Medicine, University of Porto, Porto, Portugal; ^6^Department of Gastroenterology, Centro Hospitalar São João, Porto, Portugal; ^7^Center for Drug Discovery and Innovative Medicines (MedInUp), University of Porto, Porto, Portugal; ^8^Unidade de Farmacologia Clínica, Centro Hospitalar Universitário de S. João, Porto, Portugal; ^9^Serviços Farmacêuticos, Hospital Garcia de Orta, Almada, Portugal; ^10^Instituto Português de Oncologia de Lisboa, Lisboa, Portugal; ^11^Serviços Farmacêuticos, Centro Hospitalar Universitário de Coimbra (CHUC), Coimbra, Portugal

**Keywords:** nanoparticles, liposomes, micelles, clinical evaluation, therapeutic equivalence, non-biological complex drugs (NBCDs)

## Abstract

Non-Biological Complex Drugs (NBCDs) are complex non-biological drugs comprised of large high molecular weight molecules and, often, nanoparticular structures (including liposomes and block-copolymer micelles). In the case of NBCDs, the entire complex is the active pharmaceutical ingredient and its properties cannot be fully characterized by physicochemical analysis. Moreover, the manufacturing process is fundamental in creating the correct originator product. The same is true for generic versions of the product. A recent appraisal of approval procedures for NBCDs “follow-on products” approved in Europe shows a diversity of regulatory pathways. In fact, three different abridged application procedures, under European legislation, were used: the generic application procedure of Article 10(1), the hybrid application procedure of Article 10(3), and the biosimilar application procedure of Article 10(4). Three informed consent applications via Article 10(c) from innovator companies of glatiramer acetate and sevelamer carbonate were submitted shortly after the approval of the first follow-on products. Furthermore, a number of “well-established use” applications [via Article 10(a)] were approved for iron sucrose and iron dextran complexes. In order to protect patients from the increased risks of NBCD products and NBCD follow-on products, two complementary approaches should be considered: (i) improving the regulatory procedures and their guidance documents within the pre-registration phase, and (ii) not considering interchangeability whenever clinical data is not available. With regards to the latter, the need for adequate safety and efficacy data might also include risk management programmes within post-approval pharmacovigilance actions. This, however, would depend on a risk appraisal that must be considered for individual medicinal products, based on the nature of the submitted relevant set of safety/efficacy data.

## Introduction

The current paper intends to be an expert-driven and fact-based. It was produced by a group of specialists in areas pertaining to the scientific issues, with significant experience in the development, regulatory review, and clinical use of non-biological complex drugs (NBCDs). Previous work was done by a NBCD expert group established within the framework of TI Pharma in the Netherlands, with contribution from two of the current authors (RG and BS-L).

That previous group collaborated closely with TEVA Pharmaceutical Industries Ltd, Vifor Pharma Ltd, and SANOFI, without any active interference in the outcomes of the experts' discussion and established reference texts. The group established landmark issues in the field (1, 2), which are now under further development by similar working groups, including one within the frame of the *Lygature* consortium in the Netherlands (3, 4).

The authors also acknowledge a significant amount of work produced along different collaborative frameworks, including EDQM, NYAS, and EC-JRC (5–7). Even though we focus more on the EU situation in the current text, we believe there are lessons to be learned that impact regulatory scenarios in different geographical areas and under different regulatory frameworks.

The current group of authors is comprised of experts both from academia and hospital clinic settings, looking into major challenges surrounding the clinical use of NBCDs. It discusses issues related to NBCDs, looking at state-of-the-art scientific knowledge and previously established scientific consensus and regulatory guidelines. The expert group delivered the current paper, looking into major relevant questions and giving their expert view on challenges regarding clinical and therapeutic equivalence.

## NBCDS: Definitions, Critical Analysis, Relevant Guidelines, and Regulatory Pathway Parallelism With The Case For Biosimilars

Non-Biological Complex Drugs (NBCDs) are complex non-biological drugs that comprise large high molecular weight molecules and, often, nanoparticular structures (including liposomes and block-copolymer micelles). They differ from typical small chemical molecules generally used as pharmaceutical active product ingredients and also from biotechnology-derived medicinal products (large proteins) since, for NBCDs, the entire complex is the active pharmaceutical ingredient and its properties cannot be fully characterized by physicochemical analysis. Moreover, the manufacturing process is fundamental in creating the correct originator product, as well as for the generic versions. But as in the case for biotechnology-derived medicinal products, the “product is also the process,” as it is the case for biosimilar medicinal products. NBCDs can also be characterized as complex drug products that are not biologicals (3).

In summary, NBCDs typically consist of a multitude of closely related structures, the entire complex being the active pharmaceutical ingredient. Its properties cannot be fully characterized by physicochemical analysis alone and the well-controlled robust manufacturing process is fundamental to reproduce the biofate of the innovator's medicinal product. In this category we currently include glatiramoids, iron oxide carbohydrate nanoparticles, liposomes, and polymeric micelles.

It is somewhat controversial to include drug nanocrystals in this category. In fact, in nanocrystals the size reduction delivers a very well-known increase in surface area that drives the dissolution behavior, solubility, and its dissolution rate. This is currently well-covered by the existing regulatory framework. In fact, strategies usually followed for oral delivery pharmaceutical formulations (i.e., through analysis of dissolution profiles and comparisons between originator and generic formulations), using the F2 dissolution parameter to prequalify formulations for further bioequivalence clinical trials, can suffice for most of these products. As such, in our view, for oral delivery systems, the use of formulations with nanocrystal analogs can be easily dealt with through relevant and well-established regulatory pathways, already designed for evaluating generic oral medicinal products (8–11).

“The product is the process” is the leading principle when discussing “follow-on” products of biotechnology-based products. In fact, when introducing the concept of biosimilars, as well as follow-on versions of complex systems such as NBCDs, this is the main critical factor. This means that it is the manufacturing process that ultimately defines the inherent properties and attributes of the final medicinal product, and therefore conditions of use. Trying to establish similarity while not recognizing the inherent complexity of these systems is a major error.

Critical issues arise, therefore, when trying to demonstrate equivalence between the follow-on product and the innovator product. The need for data on safety and efficacy, relevant for the therapeutic indication, means that, if “follow-on” products are accepted without supporting clinical proof of safety and efficacy (or an adequate surrogate path), their equivalence with the originator/innovator product cannot be appropriately defined. When the process of manufacturing impacts so heavily on the performance of the final product, with major consequences on its safety and efficacy, there is room for disputing the traditional generic pathway for marketing approval, bringing unacceptable uncertainty into the benefit-risk assessment (12, 13). For comparison, in the case of biotechnology drugs, the biosimilars approach, implemented in Europe after 2004, introduced an appropriate regulatory model. In the biosimilars pathway different “follow-on” categories are evaluated according to their specificities, taking into consideration their specific physicochemical and biological properties, evaluating how they impact separately on the clinical safety and efficacy determinants (14).

A basic rule to be followed, based on the much-needed protection of patients, is then to address differences on the basis of their inherent complexity of structures and delivery systems, without any oversimplification that could compromise both safety or efficacy. This major concern has been correctly addressed when developing the biosimilars pathway since 2004 (15), but does not completely address the main issues related to NBCDs.

The regulatory uncertainty related to NBCDs is a consequence of inadequately perceiving, identifying, and addressing the different layers of complexity associated with this category of medicinal products. In fact, even after regulatory approval of a different product using diverse regulatory pathways, several important issues are left open, pertaining to the major impact on decisions within their routine clinical use. Even after marketing authorization, most of these products were never assessed in an adequate comparative clinical assessment format, i.e., in complete absence of comparative clinical data on relative safety and efficacy. In most cases, no comparability studies were performed, and clinical decisions on prescription switching for patients under chronic or sub-chronic treatments are often based on biased therapeutic decisions that are not appropriately supported by clinical data. This is due to the implementation of national prescription guidelines or rulings that often inappropriately consider NBCDs as small molecule-based medicinal products, referring to INN prescription rules only. In such cases, the clinical staff, specialized MDs, and pharmacists are frequently forced to make decisions based on local constraints, not related to the availability of comparative clinical data that could otherwise shed light on safety and efficacy.

Meanwhile, for the pre-MA period (before granting marketing authorization), relevant guidance has been introduced in recent years that considers the regulatory approval of follow-on formulations of several categories of NBCDs and suggests how to compare the performance of these follow-on medicinal products with the performance of the innovative/originator medicinal product.

So far, three main categories of NBCDs have been subject to regulatory guidance, mainly looking into potential comparability of innovators and follow-on products: (i) Liposomes (with a broader set of guidance in the US-FDA for emulsions and specific liposome classes), (ii) Iron oxide carbohydrate nanoparticles, and (iii) Polymeric micelles.

*Glatiramoids* have proven to be more difficult to include in a specific and adequate regulatory guidance. The main reasons for that will be addressed separately.

For the simplification of terminology in the next sections, we will refer to “follow-on” products as “similar” products, since non-similar products will have to follow a regulatory path identical to innovators. The main issues under discussion deal with the regulatory and clinical issues that are relevant to establish adequate criteria for similarity, and pertain therefore only to the demonstration of similarity with the parent innovator medicinal product.

## Major Questions: Pre-Clinical Comparability and Clinical Relevance of Available Data

In general terms, and specifically with this issue, we need to have a clear pathway for regulation based on solid scientific data. There is no “*one-size-fits all approach*” when looking at the relevance of pre-clinical data and the availability of clinical data for marketing approval. This applies to the comparability between innovators (original/ first approved medicinal products) and a similar formulation submitted for marketing approval or, when approved, looking at its clinical comparability: is it subject, or not, to interchangeability? (16).

The previous statement might seem contradictory when looking to a well-known reality: in several European countries, follow-on/ similar formulations were previously approved without consulting, and prior to, the introduction of current regulatory guidance documents. In such cases, there was no major discussion or previous establishment of any meaningful preclinical standard for relevant clinical comparability, and certainly no debate or scientific comparison looking into potential substitution or interchangeability (17, 18).

This has been the case for a number of MA of iron oxide carbohydrate nanoparticles, but this history is in part also relevant for the discussion of tentative follow-on liposomal formulations or other categories within the general NBCD denomination umbrella. At present, glatiramoids are an even more difficult category for which we can generally state that there is no adequate preclinical comparison to establish clinical equivalence between a candidate “similar” formulation and the innovator/ original medicinal product (i.e., originator trademark Copaxone® from TEVA Pharmaceuticals Ltd). Even though the FDA dedicated extensive intramural and extramural research to the subject between 2013 and 2017 (19), follow-on products for Mylan in 2017 (and in Europe in 2018, in partnership with Synthon, almost in parallel with another European marketing authorization, through a partnership between Synthon and Alvogen) and also in 2018 for Sandoz (in partnership with Momenta Pharmaceuticals Inc.), were approved either with the use of placebo-controlled clinical data or in the GATE study against a COPAXONE arm (https://clinicaltrials.gov/ct2/show/NCT01489254).

Within the European regulatory framework, current dossier requirements have also been clarified for specific categories of NBCDs as liposomes, iron oxide carbohydrate nanoparticles, and polymeric micelles.

When looking into preclinical comparability, considering the integrated European guidance documents (reflection papers) already formulated in relation to different types of NBDCs and their similarity, common requirements were identified. These commonalities reflect the recognition by European Regulators that, by default, the proof of similarity for NBCDs deviates from that for generic's bioequivalence. The common requirements identified were: (i) Comparability of *in vitro*/*in vivo* pharmacodynamics; (ii) Pharmacokinetics and biodistribution studies (multiple time points, comparative); (iii) Biodistribution of the NBCD product in relevant organs (safety and efficacy, comparative); (iv) *in vivo* toxiclogy studies, need, and format (comparative); and (v) Need for validation of relevant and appropriate analytical methods.

In the same line, for issues relevant to the evaluation of clinically available data, the current regulatory guidance identifies gaps on knowledge and/or technologies that need to be addressed when looking into specific product/formulation to deal with: (i) insufficient knowledge on the impact of quality attributes and modifications on the activity of the products; (ii) insufficient design of studies, in order to attain the statistical power for detection of *in vivo* small differences; (iii) the need for advanced/sophisticated physicochemical analytical techniques (e.g., microscopy, imaging, etc.) for “NBCD development; and (iv) the need for using additional predictive approaches, e.g., modeling, to help to understand the impact of attribute variability on the efficacy and safety of a (similar) NBCD vs. the originator.

More recently, the US Food and Drug Administration released a Guidance to Industry paper entitled “Drug Products including biological products that contain nanomaterials” [(20) see also for Liposomes final guidance under (21)], where a number of concepts and recommendations are given for the development of innovative or follow-on/ similar products falling into the NBCD category. The guidance proposes a so called *risk-based approach*, presuming that potential risks can be anticipated based on: (i) Adequate characterization of the nanomaterial; (ii) Understanding of a nanomaterial's intended use; and (iii) Application, and how the nanomaterial attributes relate to product quality, safety, and efficacy.

Among the attributes needing characterization, as they may affect the clinical performance of the similar product, the FDA guidance document refers to: (i) Characterization of the material structure and its function; (ii) Complexity of the material structure; (iii) Understanding of the impact of physicochemical properties of the material on biological effects (e.g., the effect of particle size on pharmacokinetic parameters); (iv) Understanding the *in vivo* release mechanism based on the physicochemical properties; (v) Predictability of *in vivo* release based on established *in vitro* release methods; (vi) Physical and chemical stability; (vii) Maturity of the nanotechnology (including manufacturing and analytical methods); (viii) Potential impact of manufacturing changes, including in-process controls, and the robustness of the control strategy on critical quality attributes of the drug product; (ix) Physical state of the material upon administration; (x) Route of administration; and (xi) Dissolution, bioavailability, distribution, biodegradation, accumulation, and their predictability based on physicochemical parameters and animal studies.

This guidance applies to multiple NBCDs, like liposomal formulations, iron oxide nanoparticle formulations, or other nanoparticle-based products, and the principles could also be adapted for application to other types of NBCDs on a case-based approach.

What do we need to advance the field? What would facilitate better preclinical data with clinical relevance?

*First*, to have even better analytical tools and a better understanding of clinically meaningful parameters.

*Second*, to have adequate preclinical tests that, either *in vitro* or through adequate animal models, could deliver more clinically relevant information concerning distribution and pharmacological activity patterns and immunogenicity-related issues.

*Third*, there is a need for better ways to measure efficacy and safety in the preclinical stage and clinical use through adequate and clinically relevant biomarkers.

*Fourth*, efforts need to be supported to allow for a confidence-building environment, in which manufacturers of innovators and of follow-on candidate products can share new and relevant scientific evidence compiled by both that could help the scientific community and clinicians. Without a dialogue between manufacturers and regulators, their manufacturing processes might face severe challenges from new innovative manufacturing technologies. Additionally, health care professionals need to be integrated in an open debate in order to allow for their comprehension of the technologies involved. This is particularly relevant for the forthcoming introduction of new continuous manufacturing technologies, including process analytical technologies.

*Fifth*, to engage all stakeholders in discussions leading to science-based decisions on the interchangeability of NBCD products.

## Overview of Relevant Products and Regulatory Paths

**Figure 1:**
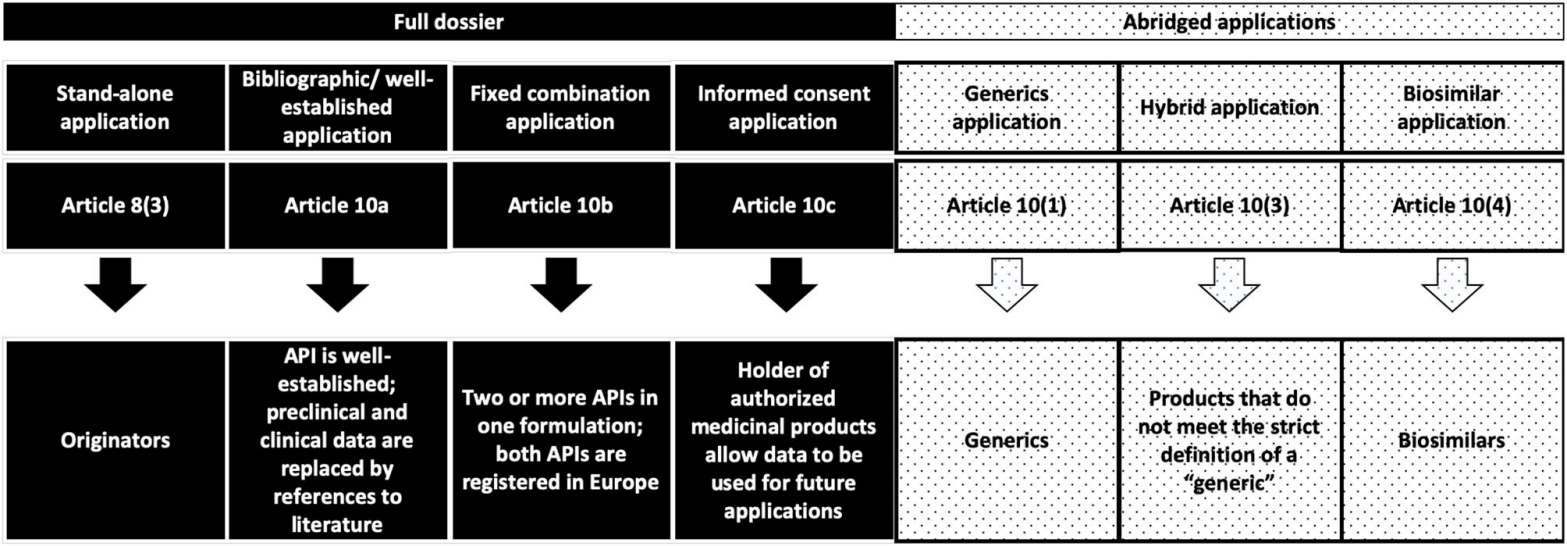
European regulatory pathways considered in the discussion for the submission of MAA related to NBCDs in the current European regulatory framework (24).

**Figure 2:**
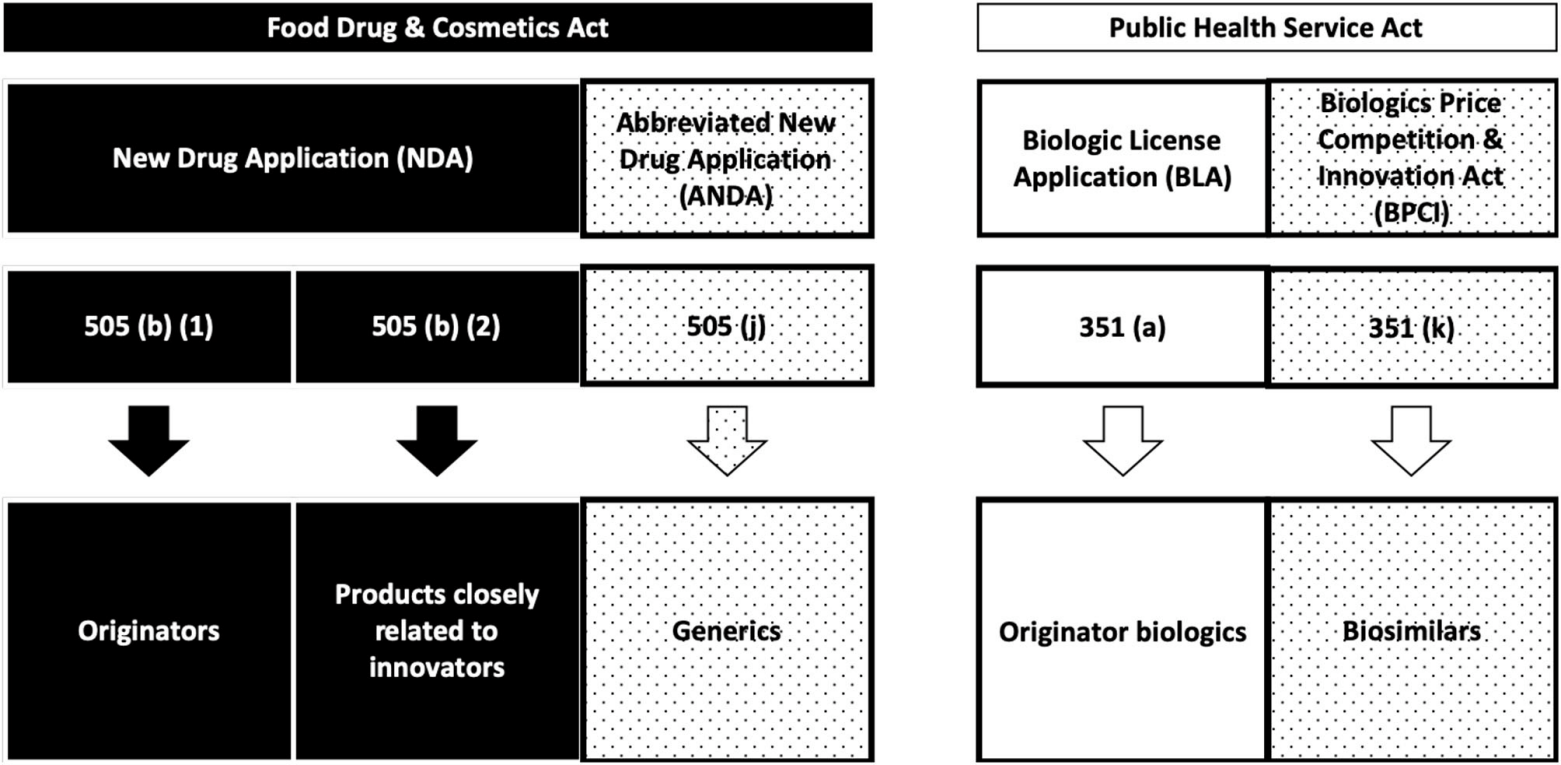
FDA regulatory pathways (24).

An overview of the current status of approval for nanopharmaceuticals can be found in the literature (22, 23) (Table 1). In general, all of the listed nanopharmaceuticals fall into the sub-categories of NBCDs mentioned before. Meanwhile, European (both EMA and national agencies, according to selected MA procedures) and FDA regulatory paths are different, bringing legal complexity and regulatory diversity for the submission strategies in the two different regions (24) (Figures 1, 2).

**Table 1:** A tentative list of currently approved nanopharmaceuticals, their indication(s), and year of approval [adapted from (22)].

**Nanotechnology**	**Name**	Drug^*^	**Indication**	Marketing approval year^**^
	Cesamet®	Nabilone	Anti-emetic	2006
	Cholib®	Fenofibrate/simvastatin	Dyslipidemia	2013
	Emend®	Aprepitant	Anti-emetic	2003
	Gris-PEG®	Griseofulvin	Antifungal	1975
	Megace ES®	Megestrol acetate	Hypercholesterolemia/ Hypertriglyceridemia	2005
Drug nanocrystals	Rapamune®	Rapamycin formulated in tablets	Immunosupression	2002
	Tricor®	Fenofibrate as nanocrystals	Hypercholesterolemia/ Hypertriglyceridemia	2004
	Triglide®	Fenofibrate as non-soluble drug delivery microparticles	Hypercholesterolemia/ Hypertriglyceridemia	2004
	Xeplion®	Paliperidone	Schizophrenia	2011
	Zypadhera®	Olanzapine	Schizophrenia	2008
	AmBisome®	Amphotericin B	Fungal infections	1990
	DepoCyt®	Cytarabine	Meningeal neoplasms	1999
	Exparel®	Bupivacaine	Anesthetic	2011
	DaunoXome®	Daunorubicin	Cancer advanced HIV-associated Kaposi's sarcoma	1996
Liposomes	Caelyx®/Doxil/®/Lipidox®	Doxorubicin HCl (pegylated)	Breast, ovarian neoplasms, multiple myeloma, Kaposi's sarcoma	1995XXXX
	Myocet®	Doxorubicin HCl	Breast neoplasms	2000
	DepoDur®	Morphine	Pain relief	2004
	Mepact®	Mifamurtide	Osteosarcoma	2009
	Visudyne®	Verteprofin	Macular degeneration/myopia	2000
	Marqibo®	Vincristine	Lymphoblastic leukemia	2013
Polymeric drugs	Copaxone®/Glatopa®	Glatiramer acetate	Multiple sclerosis	1996 2016
	VivaGel®	Dendrimer	Bacterial vaginosis	2015
	Abraxane®	Nab-Paclitaxel	Metastatic breast cancer Advanced NSCLC Metastatic pancreatic cancer Gastric cancer	2005 2012 2013 2013
	Maltofer®	Iron polymaltose	Iron deficiency	1964
Nanoparticles	Ferinject®/Injectafer®	Ferric carboxymaltose	Iron deficiency	2007
	Rienso®/FeraHeme®	Ferumoxytol	Iron deficiency	2009
	Dexferrum®	High molecular weight iron dextran	Iron deficiency	1996
	Cosmofer®	Low molecular weight iron dextran	Iron deficiency	2001
	Ferrlecit®	Iron gluconate	Iron deficiency	2009
	Monofer®	Iron isomaltoside	Iron deficiency	2009
	Venofer®	Iron sucrose	Iron deficiency	1992
	Cervarix®	Human papillomavirus (HPV) type 16L1 and 18L1 antigens	Prevention of HPV induced cancers	2007
Virus-like particles (VLPs)	Gardasil®	Major capsid protein L1 of HPV types 6-11-16-18	Prevention of HPV induced cancers	2006
	Engerix B®	Recombinant hepatitis B surface antigen	Prevention against Hepatitis B infection	1986
Virosomes	Inflexal® V	Hemagglutinin, neuraminidase antigens	Influenza	1997
	Epaxal®	Formalin inactivated hepatitis A virus (HAV)	Prevention of hepatitis A infection	1993

* *Controversial attribution of INN of the transported drug because the complex system cannot be compared directly with parent original API*.

** *When not separated it represents the approval year of first therapeutic indication*.

Several of these medicinal products have been under regulatory post-approval appraisal. On 7 December 2011, France triggered a referral under Article 31 of Directive 2001/83/EC. The CHMP was requested to give its opinion on whether the marketing authorizations for iron-containing intravenous medicinal products and associated names should be maintained, varied, suspended, or withdrawn. The procedure described in Article 32 of Directive 2001/83/EC was applicable (25) and measures related to restrictions, warnings, changes to the product information, additional pharmacovigilance activities, and risk minimization were approved. Also, propofol formulations pertaining to a nano-structured formulation which points toward the same general issues was targeted by a PRAC review (26).

A recent appraisal of approval procedures for NBCD follow-on products approved in Europe shows a diversity of regulatory pathways being followed. In fact, three different abridged application procedures—under European legislation—were followed: the generic application procedure of Article 10(1), the hybrid application procedure of Article 10(3), and the biosimilar application procedure of Article 10(4). In the latter case, there is a contrasting decision when compared with the FDA approach, where low-molecular weight heparins (LMWH) were compared as complex drugs rather than as biologics, which is how the EU approach views them. Three informed consent applications through Article 10(c) from innovator companies of glatiramer acetate and sevelamer carbonate were submitted after the approval of the first follow-on products. Furthermore, a number of well-established use applications [via Article 10(a)] were approved for iron sucrose and iron dextran complex (4).

The trend toward the hybrid application could indicate that, for certain NBCD product classes, regulatory authorities in EU member states tried to address the uncertainty of the performance of the follow-on candidate products in practice requesting additional (pre)-clinical data using the hybrid application procedure—as done with the marketing approval of Sucrofer®–through decentralized procedure, by the UK authority, in June 2018.

The different regulatory approaches taken by different MS for the approval of similar NBCDs may derive from the lack of an established classification criteria for these products (i.e., describing iron oxide nanoparticles as “aqueous solutions of iron”), which would need to be harmonized accordingly to modern evaluation methodologies and standards. While the hypothesis of the creation of a “complex hybrid” concept to harmonize with the FDA has been discussed within the EU/EMA, a legal framework for those products remains to be created. Therefore, assessment differences may emerge from the individual interpretation at the national level when product approval follows non-centralized procedures.

## Relevance of Clinical Decisions in Regulatory Paths and Mandatory Criteria for Comparability and/or Therapeutic Equivalence

There is a critical question to be addressed when decisions have to be made in a specific clinical setting: how can the similar be selected, guaranteeing therapeutic efficacy and safety for the patient, when interchangeability and substitutability of such complex drugs cannot be taken for granted?

An adequate level of clinical evidence needs to be generated if any interchangeability decision is to be taken. It cannot be generated solely based on the adoption of the common INN (in this case a misleading form of making similar what is in reality a different product). In fact, not all liposomal doxorubicin formulations can be interchanged as the same happens for iron oxide IV formulations or other macromolecular constructs of a specific API (under the same INN). In the case of NBCDs, using only an INN-based approach will increase risks for the patient.

The same goes if you consider that a regulatory marketing authorization is sufficient for interchangeability (due to the diversity of regulatory paths and the possible insufficiency of clinical data submitted before marketing authorizations).

Most of these medicinal products will have to be managed in a hospital setting, which is why the interdisciplinary pharmacotherapy committees need to consider all levels of evidence generated, focusing specifically on data related to clinical safety and efficacy comparability. The establishment of equivalence of clinical safety and efficacy for medicinal products classified as NBCDs needs the provision of adequate clinical data [(27); see also (28)].

A major question within the regulatory approval phase is to consider if a risk management plan, as a post-approval follow-up, has to be put in place at the moment of the MA (29). Looking into the level of evidence provided by most of the regulatory procedures previously adopted, we would consider it as absolutely necessary. A low level of evidence, available for comparability and eventual interchangeability, leads to inadequate safety guarantees to protect the patients.

Certainly, a better safeguard would be to use the hybrid application path within the European regulatory framework as an additional guarantee, making use of additional data that would allow evidence of safety and efficacy, based on the merits of the submitted registration file, added to existing public evidence originated by previous clinical use of quasi-similar products. In that respect, the level of similarity has to be defined in a case-by-case manner without generalization of incomparable data or products.

Our recommendations on the assessment of comparability and/or therapeutic equivalence for non-biological complex drugs are:

- comparative quality characterization based on previously established critical attributes (30);
- defining the potential impact of identified differences in the biodistribution, biological activity/efficacy, and safety using modeling and simulation approaches (31);
- pre-clinically confirming *in vitro* (potentially followed by *in vivo*) the anticipated consequences of quality-related differences (32);
- clinically confirming the preclinical findings through comparative efficacy (and safety) studies (33);
- establishing a post-marketing surveillance program for the safety of the putatively approved similar NBCD.

Moreover, for specific categories of medicinal products within the NBCDs group, our recommendations are linked to a thorough characterization of quality and of the quality attributes (34). They are key for the biodisposition and for the biological activity, efficacy, and safety. Additionally, sound and validated analytical tools for quality characterization should be made available. Also critical is the use of validated tools for efficacy and safety comparison, e.g., based on biomarkers. In that respect:

- Iron oxide nanoparticles: follow EMA and FDA guidance
- Liposomes: Follow FDA guidance
- Glatiramoids: in the absence of clear guidance, a case-based approach is recommended, where companies should ask for scientific advice from the EMA or FDA for the development of their products
- Polymeric micelles: follow EMA and FDA guidance.

In that respect, we conclude that a regulatory path adapted from biosimilars needs to be considered when looking at requirements to be imposed before any marketing authorization of a “similar” NBCDs product can be granted. This should follow a set of general guidance documents complemented by a specific set of guidance per sub-category (i.e., polymeric nanoparticles, liposomes, iron oxide nanoparticles, or glatiramoids).

A further future discussion will also need to bring on board issues related to nano-based products with new biological entities and the consequences for the enlarged community of follow-on NBCD products (35).

## Conclusions

There is no legal definition of “complex hybrids,” which might offer a more adequate regulatory path for approving NBCD (similar). That is an important path that regulatory authorities should consider. Meanwhile, in the European regulatory context, the use of hybrid applications, following Article 10(3), seems to be an adequate possibility, if supplemented with appropriate regulatory guidance on the scientific issues pertaining to establishing safety and efficacy. This is an ongoing work that needs further regulatory action from scientific committees, preparing the scenario of modifications in legal definitions within the scope of a future change in European pharmaceutical law.

When looking at therapeutic equivalence and/or clinical comparability, data from specific clinical trials comparing the innovators and follow-on/ similar products still seems to be the most prudent path. This avoids the trap of unanticipated severe side events or lack of desired efficacy that, in most cases, cannot be fully discounted, when regulatory decisions are based solely on preclinical data or simple physico-chemical characterization. Therefore, we should always exclude any generic-like path in regulatory assessments during the pre-marketing evaluation of NBCDs.

The most critical issues relate directly to the need for an appropriate frame for a decision-making procedure within the “real-life” clinical setting, when facing the choice between the innovator (original medicinal product) and its “similar” medicinal product. In fact, we are unable to implement for NBCDs the simple interchangeability decision which is normally the case after regulatory approval of generics (6) (Table 2).

**Table 2:** Selection criteria for similar nanopharmaceuticals, based on the formulary selection criteria for Biosimilars [adapted from (6)].

**Pharmaceutical quality**	**Efficacy/Safety**	**Manufacturer considerations**	**Product considerations**	**Hospital and patient factors**
Chemical composition	*Pharmacokinetics* • *Uptake* • *Distribution*	Supply reliability	Product packaging and labeling	Economic considerations • Hospital • Payer • Patient
Identity	Clinical data	History of drug shortages	Bar coding	Transition of care
Quantity	Range of indications	Supply chain security	Compatibility with CSTDs, robotics	IT and medication system changes
Pharmacopoeial specifications	Immunogenicity	Anti-counterfeit measures	Ready-to-use preparation and administration • *Stability for ready-to-use administration*	Educational requirements
*Particle size and size distribution*	Potential for therapeutic interchange	Patient assistance programs	Storage requirements	Pharmacovigilance requirements
*Particle surface characteristics*	Number of similar agents on formulary	Reimbursement support		
*Uncaptured pharmacological active substance*	Pharmacovigilance requirements	Manufacturer services, expertise		
*Storage stability*				

A decision to use mandatory centralized procedures for these products would be difficult to apply under the current legislation, after 2004. Under the current system, the use of product-class arbitration to harmonize existing products in the market could be an option, but one with major uncertainty on the final outcome.

The need to protect the patient from increased risks needs changes through two complementary approaches: (i) improving the regulatory procedures and their guidance documents in the pre-registration phase, recognizing that regulators should step in with improved regulatory guidance and better clarification of procedures; and (ii) whenever comparative clinical data is still not available (provided by appropriately designed clinical trials), the need for adequate safety and efficacy data—if interchangeability is to be considered—might also include appropriate post-marketing risk management programmes, depending on a risk appraisal that has to be considered for each individual product, based only on the nature of the submitted relevant set of safety/efficacy data.

## Author Contributions

RG and BS-L: coordination and writing and discussion of content. FM, AA, FC, and JF: writing and discussion of content. All authors contributed to the article and approved the submitted version.

## Conflict of Interest

One of the members of the current expert group (RG) also participated in the previously referred to initial NBCD expert group organized through TI Pharma, and was co-author of a publication originated from that group [(1) The AAPS Journal]. The remaining authors declare that the research was conducted in the absence of any commercial or financial relationships that could be construed as a potential conflict of interest.

## Abbreviations

API: Active Product Ingredient
BA/BE: Bioavailability/Bioequivalence
EMA: European Medicines Agency (previously EMEA, European Medicines Evaluation Agency)
EU: European Union
FDA: Food and Drugs Agency, USA
MA: Marketing Authorization
NBCDs: Non-Biological Complex Drugs
UK: United Kingdom.
